# A digitally supported home-based exercise training program and dietary protein intervention for community dwelling older adults: protocol of the cluster randomised controlled VITAMIN trial

**DOI:** 10.1186/s12877-018-0863-7

**Published:** 2018-08-14

**Authors:** Jantine van den Helder, Carliene van Dronkelaar, Michael Tieland, Sumit Mehra, Tessa Dadema, Bart Visser, Ben J. A. Kröse, Raoul H. H. Engelbert, Peter J. M. Weijs

**Affiliations:** 1 Faculty of Sports and Nutrition, Amsterdam University of Applied Sciences, Amsterdam, The Netherlands; 2 Amsterdam Center for Innovative Health Practice (ACHIEVE), Faculty of Health, Amsterdam University of Applied Sciences, Amsterdam, The Netherlands; 3 CREATE-IT Applied Research, Faculty of Digital Media and Creative Industries, Amsterdam University of Applied Sciences, Amsterdam, The Netherlands; 4 Applied Psychology, Faculty of Applied Social Sciences and Law, Amsterdam University of Applied Sciences, Amsterdam, The Netherlands; 5 Informatics Institute, University of Amsterdam, Amsterdam, The Netherlands; 6 Department of Rehabilitation, Academic Medical Center, Amsterdam, The Netherlands; 7 Department of Nutrition and Dietetics, Internal Medicine, VU University Medical Center, Amsterdam, The Netherlands; 8 Amsterdam Public Health research institute, VU University Medical Center, Amsterdam, the Netherlands

**Keywords:** Ageing, Older adults, Nutrition, Physical activity, Dietary protein intake, mHealth, Persuasive technology, Exercise, Sarcopenia

## Abstract

**Background:**

Increased physical activity and dietary protein intake are promising interventions to prevent or treat the age-related decline in physical performance in older adults. There are well-controlled exercise as well as dietary intervention studies that show beneficial effects on physical performance in older adults. In practice, however, weekly group based exercise or nutritional programs may not be as effective. To optimise these exercise programs for community dwelling older adults, a digitally supported and personalised home-based exercise training program has been designed aiming to improve physical performance in older adults. In addition, a protein intervention in combination with the training program may further improve physical performance in older adults.

**Methods:**

The VITAMIN study will be a cluster randomised controlled trial with three parallel arms. In total, 240 community dwelling older adults (≥ 55 years) participating in weekly group exercise are randomly allocated into: 1) regular weekly exercise program (Control group, *n* = 80), 2) digitally supported personalised home-based exercise training program group (VITA group, *n* = 80) and 3) digitally supported personalised home-based exercise training program group plus dietary protein counselling (VITA-Pro group, *n* = 80). The VITAMIN study aims to evaluate effectiveness of the digitally supported personalised home-based exercise training program as well as the additional value of dietary protein on physical performance after 6 months. In addition, a 12 month follow-up measurement will assess the retaining effect of the interventions. Primary outcome is physical performance measured by the Modified Physical Performance Test (M-PPT) and relevant secondary and observational outcomes include habitual physical activity and dietary intake, body composition, cognitive performance, quality of life, compliance and tablet usage. Data will be analysed by Linear Mixed Models.

**Discussion:**

To our knowledge, the VITAMIN study is the first study that investigates the impact of home-based exercise, protein intake as well as use of persuasive technology in the population of community dwelling older adults.

**Trial registration:**

NL56094.029.16 / NTR (TC = 5888; registered 03–06-2016).

## Background

The world population is ageing rapidly [1]. As society ages, the decline in physical performance among older adults dramatically increases. The decline in physical performance is associated with increased risk of falling, sarcopenia, frailty, reduction of quality of life, institutionalisation, co-morbidity, premature death and increased health care costs [2, 3]. A major cause of decline in physical performance is sedentary behaviour and a lack of physical activity [4], resulting in a strong association with the prevalence of chronic diseases [5, 6]. Interventions that stimulate physical activity, such as exercise training, seem to improve muscle mass, muscle strength and physical performance, which have a major impact on quality of life and prevention of chronic health conditions [7–10].

In the community, several group based weekly exercise programs aimed to improve physical activity in older adults. These programs, however, were not effective to improve physical performance and quality of life [11]. These outcomes may be caused by the low intensity of the exercise, the low frequency (once a week), and a lack of focus on functional exercise such as activities of daily living [ADL] [11, 12]. To optimise these exercise programs, a personalised home-based exercise training program with a higher intensity, at least twice weekly [13], and functional ADL focused exercises may be more effective to improve physical performance. This program may be further optimised by the support of technology such as a tablet computer. The latter tablet computer with application (i.e. m-health) may improve compliance, execution of the exercises and monitoring progression [14–17].

In addition to this home-based exercise training program, adequate dietary protein intake may further improve physical performance in older adults [18–20]. However, no data is available on the impact of protein intake (counselling) during personalised and digitalised home-based training programs in older adults.

Therefore, the present VITAMIN (VITal AMsterdam older adults IN the city) study aims to improve physical performance of community dwelling older adults by a modular approach:

1. A personalised home-based exercise training program to improve frequency and intensity of functional activities of daily living;
2. Digital support of the training program by a tablet computer for compliance and execution of the exercises as well as personalised coaching and monitoring;
3. Personalised dietary protein counselling to support optimal nutritional status to optimise the impact of the training program;

The objective of the VITAMIN intervention is to evaluate the 6 months effectiveness as well as 12 months follow-up retaining effect of the digitally supported home-based exercise training program as well as the additional value of dietary protein on physical performance in community dwelling older adults.

## Methods

### Study design and setting

The VITAMIN study will be a cluster randomised controlled trial with 3 parallel arms. Participants are recruited from existing weekly community based exercise groups, which will be randomly assigned (with use of computer-generated randomisation lists) as clusters to one of the following groups:

1. Regular weekly exercise program (Control group, *n* = 80),
2. Digitally supported home-based personalised exercise training program group (VITA group, *n* = 80),
3. Digitally supported home-based personalised exercise training program group plus dietary protein counselling (VITA-Pro group, *n* = 80).

**Fig. 1:**
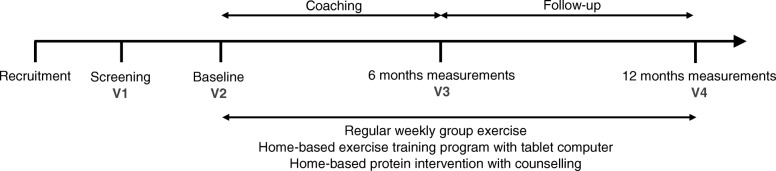
Schematic overview of study

The full study duration is 12 months. The study comprises four visits: a screening visit (V1), a post-randomisation baseline visit (V2), 6 months outcome visit (V3) and 12 month follow-up visit (V4). For the intervention groups the 12 months period includes a 6 month intervention period with personalised coaching, and a 6 month follow-up evaluation. Figure 1 provides an overview of the study.

### Subjects

We aim to recruit 240 community dwelling older adults (≥ 55 years of age). Participants from the Amsterdam region are recruited through: 1. community based weekly exercise programs provided by health care and sports organisations. 2. a mailing to 10.000 addresses of community dwelling inhabitants. All interested older adults participating in community based exercise programs are provided with an additional information package and are asked to visit the study team at their nearby community centre (V1).

The following inclusion criteria apply:

1) older than 55 years of age, 2) willingness of the general practitioner to be notified on study participation, 3) willingness to comply with the protocol in the opinion of the study physician(s), who ensures safety for the participants by screening especially for any condition, medication, or circumstance that might interfere with the study protocol, 4) ability to understand the Dutch language, 5) absence of current alcohol or drug abuse in the opinion of the investigator, 6) absence of cognitive impairment (MMSE ≤15) or 7) absence of knee or hip surgery in the last 6 months.

### Procedures

Subsequently, after obtaining a written consent, the participants will be screened (V1) at their community centre or exercise location (20–30 min). The Physical Activity Readiness Questionnaire (PAR-Q) [21], Mini-Mental State Examination (MMSE) [22] and medical and demographical data will be obtained. Afterwards, the study physicians determine the eligibility of the participants and if indicated consult the general practitioner or specialist. The eligible participants from one regular weekly exercise group will be randomised as a cluster in one of the three study groups.

Table 1 provides an overview of the location, content of assessment and duration of data collection per time point. All measurement visits (V2 - V4) take place at the Amsterdam Nutritional Assessment Center (ANAC) at the Amsterdam University of Applied Sciences (AUAS), Amsterdam, The Netherlands. For transport to the study location the participants receive a travel fee.

**Table 1:**
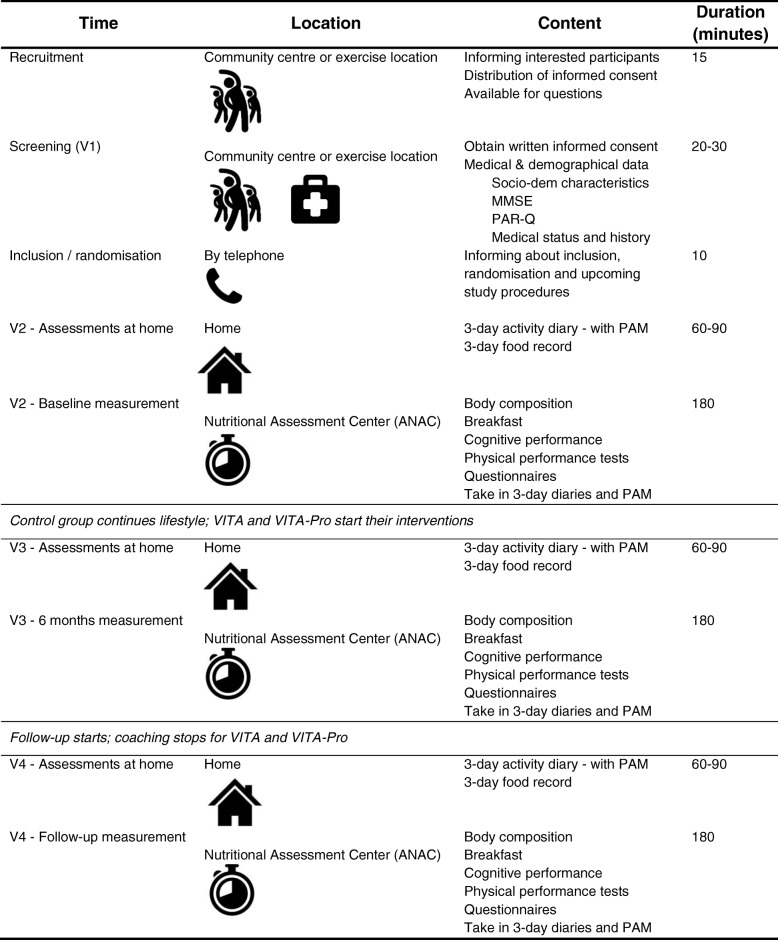
Time, location, content, duration of the VITAMIN study

Note: *ANAC* Amsterdam Nutritional Assessment Center, *PAM* physical activity monitor; *Socio-dem* Socio demographic

Before each measurement visit the participant will receive a 3-day dietary record and physical activity diary to complete at home, as well as an accelerometer to complement the physical activity diary. Subjects are instructed to be fasted prior to each measurement.

All procedures of screening, measurements and interventions are performed by an intensively trained team of students from the AUAS courses: Nutrition and dietetics, Physiotherapy, and Exercise Therapy. This multidisciplinary study team is supervised and trained according to the Dutch Medical Research Involving Human Subjects Act, ICH-GCP guidelines and coordinated by teachers/researchers: a blinded study coordinator (JH), an unblinded study assistant (CD) and two blinded teachers/researchers (SM/TD). In addition, two study physicians are responsible for the medical aspects during the study.

### Interventions

Table 2 provides an overview of the three research groups, intervention details and necessary time.

**Table 2:**
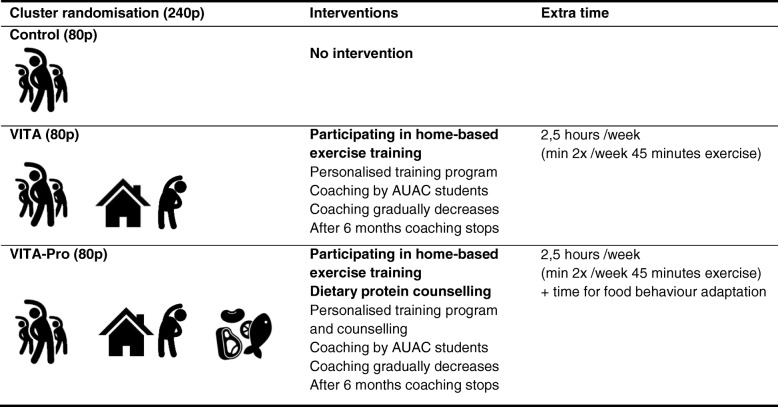
Cluster randomisation / Interventions

Note: *p* participants; *AUAC* Amsterdam University of Applied Sciences

#### Control group

In the control program, participants follow their regular weekly exercise routine without any additional intervention.

#### VITA and VITA-PRO group

Participants engage into their weekly exercise groups, and additionally they will train at least twice a week at home. This results in physical activity for three or more times a week, in order to meet the frequency of training required for effectiveness.

#### Functional exercise training

The home-based personalised exercise training program (VITAMIN-program) is a functional training program with an additional general physical activity plan. The functional exercises focus on daily activities such as climbing stairs, getting out of a chair and lifting groceries that are meaningful for participants and especially designed to improve physical performance. This VITAMIN-program is developed by the AUAS based on the Functional Training for older adults by TNO Research (The Netherlands), expertise on physiotherapy and exercise therapy, expertise on exercise training for elderly and focus group discussions with older adults and their trainers [23]. Participants formulate their own goals to get them motivated to exercise with their personalised program.

Furthermore, the recommendations for physical activity will be incorporated [24]: Physical activity > 5 days a week (at least 3), accumulate at least 30 or up to 60 min in bouts of at least 10 min each to total 150–300 min/wk., at least 20–30 min/day, on a scale of 0 to 10 for level of physical exertion, between moderate- (5–6) and vigorous- (7–8) intensity, at least 2 days a week resistance and flexibility exercises, balance exercise for individuals with mobility problems.

#### Digital support

The VITAMIN-program is supported by an application that runs on a tablet computer, designed by the AUAS and contains content to safely carry out the exercises at home. The insights from the focus group study [23] are taken into account in the user centred approach for the application development and the expertise on persuasive technology and applied psychology is used to develop the application.

#### App design and functions

The application has 5 interfaces with different functions.

1. Today: today’s program of personal exercises with video instructions and alterations of the exercises. After the exercise the participants ranks the exercise on intensity, difficulty and pleasantness.
2. Week planning; overview of the exercise training program for a week.
3. Exercises: an interface containing all exercises in the 18 categories of activities of daily living. Instructions and information about every exercise including safety aspects, necessary equipment and exercise purposes are present.
4. Coach: communication options to contact the coach (Skype, email or call).
5. Profile: A ‘wizard’ to help participants set their own program by their personal goals.

#### Dietary protein counselling

In the VITA-Pro group participants will additionally receive dietary counselling to improve their protein intake. A supervised nutritionist coaches the participant to ingest the optimal amount (1.2–1.5 g per kg body weight per day), timing (breakfast, lunch, dinner, snacks) and source of protein (high quality protein sources, e.g. dairy protein) to improve body composition and physical performance [25–27]. Participants will be advised to consume food products available in food stores that are per definition considered safe. No protein supplements will be used.

#### Coaching

The coaches will contact the participants on a weekly basis in the first 2 months for both the exercise and dietary intervention. Coaching intensity will be tailored to individual needs and will decrease over time. All counselling and coaching instructions are based on the coaching manuals, developed for the VITAMIN project.

#### Handling and storage of data and documents

Data will be collected through: 1) medical and demographical data (e.g., socio-demographic characteristics, current medicine and medical history); 2) home-assessments (e.g., diaries); 3) measurement visits (including anthropometry, cognitive function and physical performance); 4) coaching registration (reports of intervention contacts; 5) tablet data (online storage of exercise behaviour data of participants). During the assessments paper Case Report Forms (CRFs) are used as source documents. Afterwards all data is handled into online-data system Research Manager (Cloud9 Health Solutions) by double data-entry. This allows secure confidential data, protection and privacy of participants. All paper CRFs and electronic CRFs will be coded and therefore privacy of the participants will be protected. Data from the tablet computer of intervention groups is stored on an Internet server in agreement with research regulations.

### Assessments

Table 3 provides a detailed overview of the primary and secondary outcomes as well as explorative outcomes at each time point.

**Table 3:** Summary of outcome measures during study visits

	Screening V1	Baseline (T0) V2	6 months (T6) V3	12 months (T12) V4
Physical performance measurements				
Modified Physical Performance Test (M-PPT) [28]		X	X	X
6 Minute Walk Test (6MWT) [33]		X	X	X
Timed Up and Go Test (TUG) [34]		X	X	X
Short Physical Performance Battery (SPPB) [36]		X	X	X
Accelerometry with activity diary		X	X	X
Hand Grip Strength (HGS) [37]		X	X	X
Cognitive performance measurements				
Trail Making, Stroop Color Word test, Letter Fluency [38]		X	X	X
Questionnaires				
Physical Activity Readiness Questionnaire (PAR-Q) [21]	X			
Mini-Mental State Examination (MMSE) [22]	X			
3 day food record [39]		X	X	X
RAND-36 item Health Survey (SF-36) [40]		X	X	X
Geriatric Depression Scale (GDS) [41]		X	X	X
Behavioural Regulation In Exercise Questionnaire (BREQ-2) [42]		X	X	X
Body Composition				
Dual-energy X-ray absorptiometry (DXA) [45, 47]		X	X	X
Air displacement plethysmography (ADP) BodPod [44]		X	X	X
Bioelectrical impedance analysis (BIA) [48]		X	X	X
Mid upper arm muscle circumference (MUAMC) [51, 52]		X	X	X
Height / Weight / BMI [51]		X		
Explorative outcome measures				
Medical and demographical data	X	X	X	X
Tablet usage and satisfaction		X	X	X
Compliance			X	X
Sustainability				X

Note: T0 = post-randomisation baseline visit (V2), T1 = 6 months outcome visit (V3), T2 = 12 month follow-up visit (V4). *BMI* Body Mass Index

X: data will be obtained

#### Primary outcome

The primary outcome is the change in physical performance measured with the Modified Physical Performance Test (M-PPT), between baseline and 6 months [28, 29]. The M-PPT will be performed as an assessment of multiple dimensions of physical function (basic and complex activities of daily living) with different levels of difficulty. The test is well-known in elderly research [30, 31]. The test consists of 9 items [28, 32]; 1. Book lift; 2. Put on and take off a coat; 3. Pick up a coin; 4. Chair rise; 5. Turn 360°; 6. 15.2 m walk; 7. One flight of stairs; 8. Four flights of stairs; 9. Progressive Romberg test. The maximum score is 36; 4 points per item. The test takes approximately 10 to 15 min.

#### All other assessment methods

##### Physical performance tests

*6 Minute Walk Test (6 MWT).* The 6 MWT will be used as a performance-based measure and is often used for populations of healthy older adults [33]. The 6 MWT measures the distance an individual is able to walk over a total of 6 min on a hard, flat surface.

*Timed Up and Go-test (TUG test).* The TUG test will be performed as a measure of physical performance. The TUG test is a well-known test which requires walking speed, strength and balance and is often used in a frail elderly population [34, 35].

*Short physical performance battery (SPPB).* The SPPB will be performed to assess lower extremity function using measures of gait speed, standing balance, and lower extremity strength [36]. A summary performance score of 0 to 12 will be calculated by summing the scores of the tests.

*Accelerometry with activity diary.* Physical activity will be estimated with a 2 dimensional accelerometer (PAM; PE320 Digital Pedometer, Oregon Scientific, USA) and a 3-day record. Participants are requested to wear the accelerometer for 3 days (2 week days and one weekend day) and fill out their major daily physical activities for these 3 days.

*Handgrip strength (HGS).* Three consecutive measures of handgrip strength (kg) at both hands will be recorded using a hand dynamometer (Jamar, USA). The mean and maximum strength effort of the dominant hand will be collected and used as parameters of muscle functioning [37].

##### Cognitive performance tests

*Trail Making Test (TMT).* The TMT is a standardised test to assess a subject’s information processing speed and executive functioning. The score is based on the time needed to complete the test [38].

*The Stroop Color Word Test.* This test is a cognitive task to assess a subject’s information processing speed and executive functioning. The test consists out of three parts. The score is based on the time needed to complete part III [38].

*Letter Fluency Test.* The Letter Fluency test is a cognitive test in which executive function is measured. The participants are asked to generate as many words starting with specific letters within 1 min of time. The score is the total number of the correct words [38].

##### Questionnaires

*3-day food record.* The habitual food intake will be measured using a 3-day food record. This record is designed to estimate the total energy, carbohydrate, fat, protein (including timing and source) as well as micronutrients from foods which are usually consumed [39]. Food intake will be recorded on 2 week days and 1 weekend day.

*RAND-36*. Quality of life will be measured with the RAND-36. The Dutch translation of the 36-Item Short Form Health Survey (SF-36v2) is used. This questionnaire includes a set of generic, coherent, and easily administered quality-of-life measures [40].

*Geriatric Depression Scale (GDS).* This 30-item self-report assessment is used to identify depression in the population of older adults [41].

*Behavioural Regulation In Exercise (BREQ-2) Questionnaire.* This 19-item self-report assessment gains insight into motivation for exercise and regulation in this motivation. The BREQ-2 is the most widely used measure of the continuum of behavioural regulation in exercise psychology research [42].

##### Body composition

*Height, weight and BMI.* Height (m) will be measured and reported to the nearest 0.01 m using a stadiometer. Body weight (kg) will be measured and reported to the nearest 0.1 kg using the calibrated weighing scale of the BodPod without shoes or heavy clothing. Body weight and height are used to calculate BMI (kg/m^2^).

*Air Displacement Plethysmography technology (ADP) - BodPod.* The BodPod (Cosmed, USA) Gold Standard Body Composition Tracking System is an air displacement plethysmograph which uses whole-body densitometry to determine body composition (fat and fat-free mass), and can accommodate a wide range of populations [43, 44].

*Dual Energy X-ray Absorptiometry (DXA).* Body composition will be assessed by DXA. Fat free mass, fat mass and bone mineral density as well as regional measures (appendicular lean and fat mass) will be determined. Appendicular lean mass is a measure of skeletal muscle mass [45]. A DXA scan is a non-invasive procedure with very low radiation dose [46, 47].

*Bioelectrical impedance analysis (BIA).* The Tanita MC-780 MA (2015, Tanita Corporation, Japan) is used to measure Total Body Water (TBW). BIA determines the electrical impedance, or opposition to the flow of an electric current through body tissues which can be used to calculate an estimate of TBW [48]. This BIA is an 8 electrode multi-frequency segmental body composition analyser and besides TBW, visceral fat and muscle mass can be estimated.

*Waist and hip circumference.* Waist an hip circumference will be measured following the World Health Organization (WHO) protocol [49]. With both measures the waist/hip ratio can be derived [50].

*Mid upper arm circumference and triceps skinfold.* A reduction in mid upper arm muscle circumference (MUAMC) is interpreted as a loss of muscle mass. Mid upper arm circumference (MUAC) and the triceps skinfold (TSF) will be measured according to International Society for the Advancement of Kinanthropometry (ISAK) guidelines and with use of adequate equipment [51]. MUAMC and the mid upper arm muscle area (MUAMA) are calculated [52].

##### Explorative outcomes

*Socio-demographic characteristics. (self-report data)* During screening (V1) the participants report in the presence of a study team member: Age, sex, marital status, educational level, ethnicity, exercise group participation, dependency (alcohol consumption and smoking).

*Medical status and history.* Medical history and current status on illness, disabilities and diagnoses is reported by the participant during screening (V1). Also previous and current treatments and/or medications and nutritional supplements are reported. Besides this, information about current specialists or therapists is needed for the eligibility check by the study physician. This is reported in guidance of a study team member. If medical status or medication are changed during the study, this will be reported adequately. (E.g. Adverse Event or Serious Adverse Event).

*Evaluation tablet usage.* At V2 the participant fills out a short questionnaire with questions about earlier experiences with electronical devices. At V3 and V4 the new developed tablet computer behaviour will be assessed by questionnaire. Only intervention groups will receive this.

*Compliance.* Participants will be defined as compliant when ≥80% of the personalised home-based exercise training program is completed during the intervention period. This will be derived from the tablet generated database.

*Sustainability*. The home-based exercise training program will be considered sustainable when at least 80% of the participants in groups VITA and/or VITA-Pro still follow 80% of the training program as planned at baseline after 6 month follow up.

The dietary protein counselling intervention will be considered sustainable when at least 80% of the participants in VITA-Pro still ingest 80% of at least 1.2 g/kg body weight. This is measured at 12 months by evaluation with the coach and previous described measurements.

### Planned statistical analyses

The sample size calculation for the primary outcome parameter is based on the modified Physical Performance Test (M-PPT) of Villareal et al. [29] using the following estimates: Effect size: 1.7 points mean difference in physical performance (M-PPT), a standard deviation (SD) of difference of 1.75, an average cluster size of 10, with an ICC of 2.5. Using the above-mentioned estimates, a significance level (α) of 0.05 and a power of 80%, a sample size of 56 per group is estimated to be sufficient to detect a statistically significant difference in physical performance after 6 months between groups. Assuming 40% drop-out, a total number of 78 subjects is needed per group. Therefore we aim to include 80 subjects per group and in total 240 subjects.

Summary statistics (mean, median, standard deviation, and frequency distribution) will be generated for baseline characteristics. To compare the baseline characteristics between the 3 groups, one-way ANOVA or Kruskal-Wallis test will be used for continuous variables, and Chi-square or Fisher’s exact tests, for categorical variables. Changes in M-PPT, secondary and explorative outcomes will be visualised over the entire time course using mean and standard error of the mean (SEM) per treatment group.

Data will be analysed in accordance with the research questions outlined in the introduction, applying appropriate Linear Mixed Models. Differences within and between treatment groups in the outcome parameter will be analysed. Time, treatment and their interaction will be defined as fixed factors. Subject and group will be included as random factor. If imbalances occur between groups despite randomisation, the baseline values will be treated as covariates.

Detailed analyses will be stated in the Statistical Analysis Plan, which will be finalised before unblinding of the study. All statistical analyses will be performed using SPSS Statistics v22 (IBM, USA). An α of 0.05 will be used to determine statistical significance.

## Discussion

As the society ages, there is a demand for practical and innovative interventions to reduce the decline in physical performance and maintenance of muscle mass, strength and physical performance in the ageing population. Because earlier research on weekly group exercise remained ineffective on physical performance, we have developed an innovative modular approach based on a new home-based personalised training program with functional ADL based exercises. Furthermore, the dietary counselling and the overall coaching is personalised. Especially, the persuasive technology underlying our digital support improves personalisation. Besides personalisation, our digital support improves insight in the compliance and provides the opportunity to monitor progression in the training of the participant. We are interested in both the effect at 6 months coached intervention as well as 12 months follow-up retention of intervention effect.

A factor that may be considered a threat to this intervention is the inclusion of participants of regular weekly exercise groups. This approach is an easy to implement choice, since the presently existing structure and participation in the Netherlands could be updated with a more effective form of weekly exercise with added digitally supported home based exercise. However, this also requires the cooperation and time of trainers as well as consent of some involved organisations. Also, the regular exercise groups are usually indicated for older adults with some decline in physical performance however, because of the important social component of these groups also a fitter group of older adults might participate. Since, we aimed for a broad national implementation we did not exclude the more fit and able, while we intend a per protocol analysis for those older adults with declined physical functioning.

Since the digital application is developed and a small pilot study has been conducted before start of the trial, potential issues with the interventions need to be taken into account. Therefore, suboptimal performance of the digital application might contribute to a smaller effect of the intervention on physical performance.

The findings of this study are relevant for future developments in health and community care and professional education. Also the designed technology provides new opportunities for application designers. The VITAMIN study will provide novel insights in the impact of home-based exercise, protein intake and use of technology in the population of community dwelling older adults.

## Abbreviations

6 MWT: 6 Minute Walk Test
ADL: Activities of daily living
ADP: Air displacement plethysmography
ANAC: Amsterdam Nutritional Assessment Center
AUAS: Amsterdam University of Applied Sciences
BIA: Bioelectrical impedance analysis
BMI: Body mass index
BREQ-2: Behavioural regulation in exercise questionnaire
CRF: Case report form
DXA: Dual-energy X-ray Absorptiometry
GDS: Geriatric depression scale
HGS: Hand grip strength
ICC: Intraclass correlation coefficient
ICH-GCP: International Conference on Harmonisation’s (ICH) Guideline for Good Clinical Practice (GCP)
METC: Medical ethics committee
MMSE: Mini-mental state examination
M-PPT: Modified physical performance test
MUAMA: Mid upper arm muscle area
MUAMC: Mid upper arm muscle circumference
PAM: Physical activity accelerometer
PAR-Q: Physical activity readiness questionnaire
RAND-36: RAND-36 item Health Survey (SF-36)
SD: Standard deviation
SPPB: Short physical performance battery
SPSS: Statistical package for the social sciences
TBW: Total body water
TSF: Triceps skin fold
TUG: Timed up and go test
VITAMIN: VITal AMsterdam older adults IN the city
VUmc: VU University Medical Center
WHO: World Health Organization
